# Supplementary material for: Multi‐dataset identification of innovative feature genes and molecular mechanisms in keratoconus
Source: J Cell Mol Med. 2024 Sep 19;28(18):e70079. doi: 10.1111/jcmm.70079 (PMC11412914; doi:10.1111/jcmm.70079)
Supplement: Supplementary file 2 — Material S2. [file JCMM-28-e70079-s002.zip › 7.GSEA analysis/1.ARL11/KEGG_HEDGEHOG_SIGNALING_PATHWAY.html]

Details for gene set KEGG\_HEDGEHOG\_SIGNALING\_PATHWAY[GSEA]

|  || Dataset | ARL11.group.cls#h\_versus\_l |
| Phenotype | group.cls#h\_versus\_l |
| Upregulated in class | l |
| GeneSet | KEGG\_HEDGEHOG\_SIGNALING\_PATHWAY |
| Enrichment Score (ES) | -0.32753617 |
| Normalized Enrichment Score (NES) | -1.0464686 |
| Nominal p-value | 0.39376217 |
| FDR q-value | 1.0 |
| FWER p-Value | 0.998 |
Table: GSEA Results Summary

  

Fig 1: Enrichment plot: KEGG\_HEDGEHOG\_SIGNALING\_PATHWAY      
 Profile of the Running ES Score & Positions of GeneSet Members on the Rank Ordered List

  

| SYMBOL | TITLE | RANK IN GENE LIST | RANK METRIC SCORE | RUNNING ES | CORE ENRICHMENT || 1 | ZIC2 | na | 3063 | 0.238 | -0.0518 | No |
| 2 | LRP2 | na | 3193 | 0.232 | -0.0199 | No |
| 3 | WNT5A | na | 3234 | 0.230 | 0.0143 | No |
| 4 | CSNK1G1 | na | 4283 | 0.190 | 0.0133 | No |
| 5 | WNT9A | na | 5395 | 0.155 | 0.0050 | No |
| 6 | WNT10B | na | 6703 | 0.119 | -0.0145 | No |
| 7 | IHH | na | 7016 | 0.112 | -0.0064 | No |
| 8 | SMO | na | 7558 | 0.101 | -0.0065 | No |
| 9 | PTCH1 | na | 7694 | 0.098 | 0.0046 | No |
| 10 | GAS1 | na | 7791 | 0.096 | 0.0167 | No |
| 11 | GLI3 | na | 7876 | 0.095 | 0.0288 | No |
| 12 | BMP5 | na | 8473 | 0.083 | 0.0244 | No |
| 13 | SUFU | na | 8482 | 0.083 | 0.0370 | No |
| 14 | WNT3A | na | 8640 | 0.080 | 0.0448 | No |
| 15 | RAB23 | na | 9230 | 0.071 | 0.0387 | No |
| 16 | BMP8B | na | 9337 | 0.070 | 0.0464 | No |
| 17 | BMP8A | na | 9599 | 0.066 | 0.0489 | No |
| 18 | PRKX | na | 9610 | 0.066 | 0.0587 | No |
| 19 | CSNK1A1 | na | 9614 | 0.066 | 0.0687 | No |
| 20 | WNT8A | na | 12464 | 0.032 | -0.0086 | No |
| 21 | WNT1 | na | 12803 | 0.029 | -0.0139 | No |
| 22 | GLI2 | na | 15544 | 0.007 | -0.0919 | No |
| 23 | WNT3 | na | 16162 | 0.003 | -0.1093 | No |
| 24 | CSNK1G2 | na | 16589 | -0.000 | -0.1215 | No |
| 25 | WNT10A | na | 16653 | -0.001 | -0.1231 | No |
| 26 | BTRC | na | 16988 | -0.003 | -0.1323 | No |
| 27 | HHIP | na | 17366 | -0.006 | -0.1423 | No |
| 28 | WNT4 | na | 18621 | -0.014 | -0.1762 | No |
| 29 | FBXW11 | na | 18745 | -0.015 | -0.1774 | No |
| 30 | PRKACB | na | 18969 | -0.017 | -0.1813 | No |
| 31 | STK36 | na | 19690 | -0.022 | -0.1987 | No |
| 32 | WNT5B | na | 19914 | -0.023 | -0.2016 | No |
| 33 | GSK3B | na | 20728 | -0.029 | -0.2205 | No |
| 34 | BMP7 | na | 20858 | -0.030 | -0.2196 | No |
| 35 | CSNK1D | na | 21052 | -0.032 | -0.2203 | No |
| 36 | WNT2 | na | 22576 | -0.046 | -0.2572 | No |
| 37 | BMP6 | na | 22607 | -0.046 | -0.2510 | No |
| 38 | PRKACA | na | 23665 | -0.058 | -0.2726 | No |
| 39 | CSNK1A1L | na | 23889 | -0.061 | -0.2697 | No |
| 40 | CSNK1G3 | na | 24216 | -0.065 | -0.2691 | No |
| 41 | PRKACG | na | 25752 | -0.087 | -0.3001 | No |
| 42 | WNT7A | na | 26706 | -0.103 | -0.3118 | Yes |
| 43 | BMP4 | na | 27068 | -0.110 | -0.3054 | Yes |
| 44 | WNT7B | na | 27789 | -0.125 | -0.3069 | Yes |
| 45 | WNT16 | na | 28062 | -0.132 | -0.2944 | Yes |
| 46 | PTCH2 | na | 28260 | -0.138 | -0.2789 | Yes |
| 47 | DHH | na | 29036 | -0.157 | -0.2771 | Yes |
| 48 | WNT6 | na | 29533 | -0.173 | -0.2648 | Yes |
| 49 | BMP2 | na | 30615 | -0.208 | -0.2640 | Yes |
| 50 | WNT9B | na | 30689 | -0.211 | -0.2338 | Yes |
| 51 | WNT8B | na | 31805 | -0.253 | -0.2272 | Yes |
| 52 | SHH | na | 33891 | -0.404 | -0.2254 | Yes |
| 53 | WNT11 | na | 33983 | -0.417 | -0.1639 | Yes |
| 54 | GLI1 | na | 34157 | -0.445 | -0.1006 | Yes |
| 55 | WNT2B | na | 34700 | -0.762 | 0.0008 | Yes |
Table: GSEA details [plain text format]

  

Fig 2: KEGG\_HEDGEHOG\_SIGNALING\_PATHWAY      
 Blue-Pink O' Gram in the Space of the Analyzed GeneSet

  

Fig 3: KEGG\_HEDGEHOG\_SIGNALING\_PATHWAY: Random ES distribution      
 Gene set null distribution of ES for **KEGG\_HEDGEHOG\_SIGNALING\_PATHWAY**
